# Reported time savings and telephone consultation use in polish primary care: a national random sample survey

**DOI:** 10.3389/fpubh.2026.1940990

**Published:** 2026-09-25

**Authors:** Agata Olearczyk, Katarzyna Byszek-Stevens, Jan Zwierzchowski, Anna Marszałek, Anna Gawrońska, Katarzyna Luchowska, Małgorzata Chmielewska, Barbara Więckowska

**Affiliations:** 1 Collegium of Socio-Economics, SGH Warsaw School of Economics, Warsaw, Poland; 2 Collegium of Economic Analyses, SGH Warsaw School of Economics, Warsaw, Poland

**Keywords:** access, health economics, health education, health equity (MeSH), primary health care

## Abstract

**Introduction:**

Teleconsultations may reduce travel and time burdens in post-pandemic primary care, but evidence on whether reported time savings are associated with their usage remains limited in Central Europe.

**Methods:**

A cross-sectional computer-assisted personal interviewing (CAPI) survey using a random sampling design was conducted among adults in Poland from November 2025 to January 2026 (*N* = 1,004). Logistic regression models examined telephone consultation use among 798 respondents who either had contacted primary care or needed to do so in the previous 12 months. The complete-case sample sizes were 789 in Models 1–4 and 736 in Models 5–6.

**Results:**

Reported time savings were not significantly associated with use in Models 1–4. In Model 5, which simultaneously included residence-size controls and voivodeship (NUTS 2 region) indicators and used the smaller complete-case sample, the adjusted odds ratios were as follows: 3.03 for less than 1 h, 3.91 for 1–2 h, and 4.41 for 3 h or more (all *p* < 0.01). The predicted probabilities ranged from 26.3% for no savings to 53.6% for 3 h or more. Households with average or poor financial situations were associated with lower odds of teleconsultation use compared to those in a very good financial situation.

**Conclusion:**

In fully adjusted models including place-of-residence controls and voivodeship fixed effects, perceived time savings were independently associated with higher odds of teleconsultation use in primary care in Poland. However, this association varied depending on model specifications. Therefore, policy efforts should address regional differences in digital and informational barriers contributing to inequalities in utilization.

## 1 Introduction

The demand for accessible and efficient healthcare has accelerated the adoption of telemedicine, particularly synchronous teleconsultations (1–3). Advances in information and communication technologies have enabled clinicians and patients to interact without the need for physical presence (4, 5). Although telemedicine has a longer history, its adoption expanded rapidly during the COVID-19 pandemic and has remained an important component of post-pandemic service delivery (6–8).

Teleconsultation is a synchronous, real-time clinical interaction between a health professional and a patient, most commonly by telephone or video (9–11). It can facilitate follow-up care, management of stable chronic conditions, discussion of test results, prescription renewal, referral, preventive counseling, mental health support, and triage when an in-person examination is not required (1, 12, 13). National legislation, reimbursement rules, and professional guidance determine when remote care is appropriate and how it should be organized (14–16).

From a patient’s perspective, teleconsultations can reduce travel time, waiting times, and time away from work or other activities. They may also improve access for people with mobility limitations or those living far from healthcare services (11, 15, 17). However, there are potential disadvantages including the absence of physical examination, privacy concerns, communication barriers, and unequal access to devices, connectivity, or digital skills (10, 11, 18, 19). These trade-offs make teleconsultation use a public health and equity issue rather than solely a service-delivery choice.

International evidence suggests that the uptake of remote primary care may vary across socioeconomic and geographic groups. European studies have reported socioeconomic inequalities in the use of digital primary care services, and the associations between place of residence and urbanicity have varied across settings (20–22). These findings indicate that the availability of remote care does not necessarily translate into equal utilization across population groups and highlight the importance of examining these patterns within individual healthcare systems.

Poland is a European Union member state with a universal social health insurance system in which the National Health Fund (Narodowy Fundusz Zdrowia; NFZ) serves as the principal public purchaser (23). Primary care acts as the usual first point of contact for patients and provides initial assessment and management, referrals, prevention, and ongoing care.

Polish law permitted the provision of selected health services using information and communication technologies before the COVID-19 pandemic, but the use of these services expanded rapidly in 2020 (24–27). In primary care, telephone consultations may be used for issues that can be managed without direct physical examination, including specific follow-up and administrative aspects of care. The organizational standards for teleconsultations in primary healthcare, as set out in the consolidated text published in 2022, specify requirements concerning patient identification, confidentiality, documentation, and the circumstances in which direct contact with the patient is required (28, 29). Current patient guidelines also identify circumstances in which telephone advice may be suitable and when in-person assessment is necessary (28). According to this regulation, direct contact, rather than a teleconsultation, is required under the following circumstances: when a patient consults their primary care physician, nurse, or midwife designated in their declaration of choice for the first time; when a chronic condition has deteriorated, or existing symptoms have changed; when malignancy is suspected; or when the patient or their legal guardian does not consent to a teleconsultation. Direct contact is also mandatory for children under 6 years of age, with specified exceptions for follow-up care, scheduled vaccinations, and preventive health examinations (28, 29).

Time saving is frequently reported as a benefit of teleconsultations; however, population-level evidence from Poland on the relationship between reported time savings and the actual use of telephone consultation remains limited (15, 17, 18, 30, 31). This question is relevant to post-pandemic primary care policy because time savings may differ by socioeconomic groups or regions. The study, therefore, examined the association between reported or anticipated time savings and telephone consultation use among adults in Poland and assessed whether this association persisted across sequentially adjusted models.

## 2 Materials and methods

### 2.1 Study design and population

A national cross-sectional survey of adults aged 18 years or older in Poland was conducted between November 2025 and January 2026 using a stratified random sampling design. Fieldwork was conducted using computer-assisted personal interviewing (CAPI), with computer-assisted telephone interviewing (CATI) and computer-assisted web interviewing (CAWI) available as supplementary modes. PBS Sp. z o.o., a Polish social and market research agency, conducted fieldwork under contract with the study team (32). The study team developed the concept, methodology, and questionnaire. Before finalization, the draft questionnaire was reviewed by employees of the Institute of Social Economy at SGH Warsaw School of Economics, who provided written feedback on its content and wording. A six-person expert focus group—comprising two medical doctors, two sociologists, a public policy researcher, and an economist—was subsequently convened in October 2025 to discuss clarity, simplifications, and any potential ambiguities. The revised instrument was piloted during a soft-launch phase before the main fieldwork commenced; no structural changes were required. As no previously validated instrument for measuring perceived time savings associated with primary care teleconsultations was identified, all items were developed specifically for this study. The final questionnaire comprised approximately 30 closed-ended or multiple-choice items organized into five sections—primary care experience, teleconsultation experience, clinical vignettes, perceived advantages and disadvantages, and financial aspects—alongside 10 sociodemographic items. Several items used branching logic and were administered only to relevant subgroups. The protocol was approved by the Ethics Committee at the Warsaw School of Economics (decision No. 28/2025, 3 November 2025), and all participants provided informed consent before participation.

A stratified sampling design was used to obtain national geographic coverage. The National Official Register of the Territorial Division of the Country (TERYT) was used to define territorial units for stratification (33). The target net sample was at least 1,000 respondents, and 1,004 respondents completed the survey. The questionnaire comprised approximately 30 closed-ended or multiple-choice questions, including 4 clinical vignettes and sociodemographic items.

### 2.2 Measures

The study-specific questionnaire was developed based on a narrative review of existing evidence regarding patient perspectives, effectiveness, and implementation barriers related to teleconsultation and primary care (11, 15, 18, 26). The primary focus was on the reported or expected time savings associated with using a telephone consultation instead of an in-person primary care visit. All respondents were asked a single question: “How much time do you usually save (or could you save) by using a telephone consultation with a primary care physician instead of an in-person visit?” This format indicated that respondents did not answer different questions based on their previous experiences with telephone consultations; instead, the question aimed to capture both reported and anticipated time savings in a single measure. The response categories were as follows: no savings, less than 1 h, 1–2 h, and 3 h or more. A separate five-point Likert item assessed respondents’ general agreement that teleconsultations save time, and another item assessed their agreement that teleconsultations save money.

The binary outcome was the self-reported use of a telephone consultation with a primary care physician during the previous 12 months. Primary care refers to services provided by a general practitioner or family physician in the publicly funded healthcare system. The analysis also included self-rated health, age, sex, marital status, presence of children younger than 18 years in the household, education, household financial situation, employment status, size of place of residence, and voivodeship.

### 2.3 Statistical analysis

Analyses were conducted in Stata 19 (StataCorp, College Station, TX, United States). Table 1 presents unweighted counts and percentages. The analysis-eligible sample comprised respondents who either reported having contact with primary care or indicated a need for primary care during the previous 12 months (*n* = 798). Respondents who reported neither contact nor need were excluded. Complete-case estimation yielded 789 observations in Models 1–4 and 736 observations in Models 5–6.

**Table 1:** Characteristics of the study population: total sample and analysis-eligible sample.

Characteristic	Category	Total sample, *N* = 1,004	Analysis-eligible sample, *N* = 798
Age group			
	Under 25	67 (6.7)	31 (3.9)
	25–34	152 (15.1)	95 (11.9)
	35–44	224 (22.3)	167 (20.9)
	45–54	191 (19.0)	159 (19.9)
	55–64	218 (21.7)	198 (24.8)
	65 or older	152 (15.1)	148 (18.5)
Sex			
	Female	582 (58.0)	482 (60.4)
	Male	422 (42.0)	316 (39.6)
Self-rated health			
	Very good	305 (30.4)	176 (22.1)
	Good	435 (43.3)	363 (45.5)
	Fair	205 (20.4)	202 (25.3)
	Poor	51 (5.1)	50 (6.3)
	Very poor	7 (0.7)	7 (0.9)
	Refusal	1 (0.1)	0 (0.0)
Education			
	Primary or lower secondary	28 (2.8)	22 (2.8)
	Basic vocational	261 (26.0)	206 (25.8)
	Secondary	520 (51.8)	412 (51.6)
	Post-secondary	45 (4.5)	40 (5.0)
	Higher education	150 (14.9)	118 (14.8)
Place of residence			
	Rural area	404 (40.2)	314 (39.3)
	Town with fewer than 20,000 inhabitants	64 (6.4)	58 (7.3)
	Town with 20,000–99,999 inhabitants	249 (24.8)	197 (24.7)
	City with 100,000–499,999 inhabitants	153 (15.2)	124 (15.5)
	City with 500,000 or more inhabitants	134 (13.3)	105 (13.2)
Employment status			
	Employed under an employment contract	622 (62.0)	483 (60.5)
	Civil law or temporary contract	28 (2.8)	19 (2.4)
	Self-employed	41 (4.1)	31 (3.9)
	Unemployed	11 (1.1)	5 (0.6)
	Retired (old age pension)	222 (22.1)	210 (26.3)
	Receiving a disability pension	13 (1.3)	12 (1.5)
	Student	48 (4.8)	25 (3.1)
	Homemaker	19 (1.9)	13 (1.6)
Household financial situation			
	Very good	146 (14.5)	110 (13.8)
	Rather good	527 (52.5)	429 (53.8)
	Average	279 (27.8)	214 (26.8)
	Rather poor	46 (4.6)	39 (4.9)
	Very poor	6 (0.6)	6 (0.8)
	Refusal	0 (0.0)	0 (0.0)
Marital status			
	Single	169 (16.8)	107 (13.4)
	Informal partnership	85 (8.5)	56 (7.0)
	Married	726 (72.3)	613 (76.8)
	Other	24 (2.4)	22 (2.8)
Children under 18 in the household			
	Yes	299 (29.8)	223 (27.9)
	No	705 (70.2)	575 (72.1)
Primary care contact during the previous 12 months			
	Yes	744 (74.1)	744 (93.2)
	No, but primary care was needed	54 (5.4)	54 (6.8)
	No contact and no reported need	206 (20.5)	0 (0.0)
Frequency of primary care use among respondents reporting contact (*n* = 744)			
	Once a year	121 (16.3)	121 (16.3)
	Once every 6 months	241 (32.4)	241 (32.4)
	Every few months	293 (39.4)	293 (39.4)
	Once a month	76 (10.2)	76 (10.2)
	More than once a month	10 (1.3)	10 (1.3)
	Do not know	3 (0.4)	3 (0.4)
Usual form of primary care contact among respondents reporting contact (*n* = 744)			
	Only in-person consultations	462 (62.1)	462 (62.1)
	Mainly in-person consultations	239 (32.1)	239 (32.1)
	Mainly remote consultations	43 (5.8)	43 (5.8)
Travel time to the primary care facility among respondents reporting contact (*n* = 744)			
	Up to 15 min	401 (53.9)	401 (53.9)
	16–30 min	263 (35.3)	263 (35.3)
	31–45 min	63 (8.5)	63 (8.5)
	46–60 min	16 (2.2)	16 (2.2)
	More than 60 min	1 (0.1)	1 (0.1)

Values are *n* (%). Percentages for age, sex, health, education, residence, employment, financial situation, marital status, children, and primary care contact are calculated using the corresponding column total. Percentages for frequency, usual form, and travel time are calculated among the 744 respondents who reported primary care contact. The analysis-eligible sample included respondents who reported contact or a need for primary care during the previous 12 months. Employment and marital status labels were reconciled with the category order in the supplied six-model regression output; authors should confirm them against the questionnaire codebook before submission.

Binary logistic regression models were estimated sequentially. Model 1 included reported time savings. Model 2 included self-rated health. Model 3 included age, sex, marital status, and the presence of children in the household. Model 4 included education, household financial situation, and employment status. Model 5 included size of place of residence and voivodeship indicators. Model 6 additionally adjusted for general perceptions that teleconsultations save time and money. The results are reported as odds ratios with robust standard errors clustered at the sampling-cluster level. Two-sided *p*-values below 0.05 were considered statistically significant; *p*-values below 0.10 indicate weak statistical evidence. Predicted probabilities were calculated from Model 5.

## 3 Results

The total sample included 1,004 respondents. The analysis-eligible sample included 798 respondents: 744 who reported primary care contact and 54 who reported a need for primary care without contact in the previous 12 months. Compared to the total sample, the analysis-eligible sample contained fewer respondents younger than 25 years (3.9% vs. 6.7%) and more respondents aged 65 years or older (18.5% vs. 15.1%). Distributions by sex, education, and place of residence were similar (Table 1).

Models 1–4 were estimated using 789 complete cases. In Model 1, none of the reported time savings categories were statistically significant: The odds ratios were 0.84 for less than 1 h, 1.50 for 1–2 h, and 1.18 for 3 h or more. The associations remained statistically non-significant after adjusting for health, demographic, socioeconomic, and employment characteristics in Models 2–4 (Table 2).

**Table 2:** Logistic regression models of telephone consultation use and reported time savings (odds ratios).

Predictor	(1)	(2)	(3)	(4)	(5)	(6)
*Dependent variable: used a telephone consultation in primary care during the previous 12 months (yes/no)*						
Reported time savings (reference: no savings)						
< 1 h	0.840	0.818	0.761	0.780	3.032***	3.154***
	(0.217)	(0.213)	(0.205)	(0.216)	(1.244)	(1.333)
1–2 h	1.501	1.480	1.427	1.376	3.907***	4.134***
	(0.388)	(0.386)	(0.381)	(0.380)	(1.596)	(1.752)
3 h or more	1.180	1.152	1.017	1.063	4.408***	4.866***
	(0.391)	(0.386)	(0.350)	(0.383)	(2.196)	(2.525)
Constant	0.947	1.052	0.697	2.515	0.588	0.384
	(0.220)	(0.280)	(0.312)	(2.601)	(0.773)	(0.530)
N	789	789	789	789	736	736
Pseudo *R*^2^	0.013	0.017	0.033	0.066	0.202	0.218
Health status controls	No	Yes	Yes	Yes	Yes	Yes
Demographic controls	No	No	Yes	Yes	Yes	Yes
Socioeconomic/employment controls	No	No	No	Yes	Yes	Yes
Residence size controls	No	No	No	No	Yes	Yes
Voivodeship indicators	No	No	No	No	Yes	Yes
Perceived time/money savings	No	No	No	No	No	Yes

Odds ratios are reported, with robust standard errors clustered at the sampling-cluster level in parentheses. The analysis-eligible sample included respondents who reported primary care contact or a need for such contact during the previous 12 months. Reference category for reported time savings: No time savings. Model 5 added residence size controls and voivodeship indicators; Model 6 additionally adjusted for perceived time and money savings. *p < 0.10; **p < 0.05; ***p < 0.01. Full covariate estimates are provided in Supplementary Table S1.

Model 5 included residence-size controls and voivodeship indicators and was estimated using a smaller complete-case sample (*n* = 736). In this specification, all reported time savings categories were associated with higher odds of using telephone consultations compared to no reported savings. Specifically, the odds ratios were as follows: for less than 1 h, the adjusted OR was 3.03 (robust SE = 1.24); for 1–2 h, the adjusted OR was 3.91 (robust SE = 1.60); and for 3 h or more, the adjusted OR was 4.41 (robust SE = 2.20); all *p* < 0.01. Model 6 produced similar estimates after including general perceptions of time and money savings (adjusted ORs = 3.15, 4.13, and 4.87, respectively; all *p* < 0.01). As Model 5 simultaneously included two sets of controls and changed the estimation sample, the coefficient shift cannot be attributed to voivodeship indicators alone.

A same-sample sensitivity check was performed by re-estimating Model 4 using the complete-case sample in Models 5 and 6 (*N* = 736). The odds ratios increased to 1.84 for less than 1 h, 2.91 for 1–2 h, and 2.51 for 3 h or more; the last two categories became statistically significant, while the evidence for less than 1 h was weaker. This suggests that missing data-related sample restriction contributed to the change in estimates but did not fully explain it, as the odds ratios remained lower than those in Model 5.

Predicted probabilities from Model 5 were 26.3% (approximate 95% CI 14.5–38.1%) for no reported savings, 46.4% (41.1–51.7%) for less than 1 h, 51.3% (46.4–56.2%) for 1–2 h, and 53.6% (42.2–65.0%) for 3 h or more (Table 3). The largest absolute difference was between no reported savings and any reported savings; the increase across the three positive time savings categories was smaller.

**Table 3:** Predicted probabilities of telephone consultation use by reported time savings (model 5).

Reported time savings	Predicted probability	Robust SE	Approximate 95% CI
No time savings	0.263	0.060	0.145–0.381
< 1 h	0.464	0.027	0.411–0.517
1–2 h	0.513	0.025	0.464–0.562
3 h or more	0.536	0.058	0.422–0.650

Predicted probabilities and robust standard errors are reproduced from the supplied margins output for Model 5 (*N* = 736). Approximate 95% confidence intervals were calculated as estimate +/− 1.96 x the displayed standard error; small differences from intervals calculated using unrounded estimates are possible.

In Model 5, poor self-rated health was associated with higher odds of use compared to very good health (adjusted OR = 3.21, *p* < 0.05). Households with average or poor financial situations were associated with lower odds of teleconsultation use compared to those in a very good financial situation (adjusted OR = 0.38 and 0.22, respectively; both *p* < 0.01). Residence in a city with more than 500,000 inhabitants was associated with lower odds in Model 5 (adjusted OR = 0.53, *p* < 0.05), but the estimate was not statistically significant in Model 6. Receiving a disability pension was associated with higher odds of teleconsultation use in Models 5 and 6, although the estimates were imprecise. In Model 6, general agreement that teleconsultations save time showed only weak evidence of an association (*p* < 0.10), and perceived money savings were not statistically significant. Full covariate estimates are reported in Supplementary Table S1.

## 4 Discussion

In this national cross-sectional survey, reported or anticipated time savings were associated with the use of telephone consultations only in Models 5 and 6. The adjusted odds ratios in Model 5 ranged from 3.03 to 4.41, and the predicted probabilities ranged from 26.3% for no reported savings to 53.6% for 3 h or more. Lower household financial status was also associated with lower use of these services. These findings identify potentially important patient-perceived and equity dimensions of post-pandemic remote primary care.

The direction of the fully adjusted estimates is consistent with evidence that remote care can reduce waiting times, travel time, and time away from usual activities. Capodici et al. reported reductions in outpatient waiting times across the settings included in their systematic review and quantitative analysis (30). In Queensland, Australia, Snoswell et al. estimated that 30,149 telehealth consultations avoided approximately 9.6 million km of travel and 27,068 days away from usual activities, with an estimated societal productivity benefit of AUD 304 per consultation (17). Reviews of primary care teleconsultations have similarly identified convenience and time saving as patient-valued benefits while emphasizing the importance of appropriate clinical selection, infrastructure, and professional support (11, 15). The present study adds evidence on the association between a quantified, self-reported benefit and use in Poland; however, it does not establish temporal order because the use of these services may also shape respondents’ perceptions of the saved time.

The sharp change between Models 4 and 5 is analytically important. Model 5 included both residence size variables and voivodeship indicators, while the sample size decreased from 789 to 736 due to missing covariate data. This observed change may therefore reflect regional adjustment, residence size adjustment, selection into the complete-case sample, or a combination of these factors. To partially address this, Model 4 was re-estimated using the same complete-case sample in Models 5 and 6 (*N* = 736). This sensitivity check indicated that missing data-related sample restriction contributed to the change in estimates but did not fully explain it—the odds ratios remained substantially lower than those in Model 5, pointing to territorial adjustments as an additional source of the shift. A stronger causal or structural interpretation would still require separate models in which residence and voivodeship terms are added sequentially, together with a reported joint test of the regional indicators. Evidence from Portugal also suggests that awareness of available remote options, information and communication technology investment, and professional training can influence the uptake of these services; however, the study did not show a simple, uniform digital-divide pattern (20).

The emergence of the association after adding residence-size controls and voivodeship indicators suggests that the territorial context is significant for the relationship between perceived time savings and telephone consultation use. This is plausible given that the implementation of telemedicine in Poland has been shaped by regulatory, organizational, infrastructural, and provider-level factors, which may vary across regions and types of residence. Regional differences in the availability, organization, or digital maturity of primary care services may therefore have masked the individual-level association in less-adjusted models. The association between lower household financial status and reduced use of telephone consultations warrants attention because publicly funded access does not remove informational, technological, or confidence-related barriers. In Sweden, Wilkens et al. found there was a pro-rich inequality in the use of digital primary care contacts, even after adjusting for healthcare needs, and office visits were more frequent among lower-income patients (21). Digital health equity frameworks emphasize that access depends not only on formal availability but also on digital determinants such as connectivity, literacy, usability, trust, and how well services fit individuals’ circumstances (19). These mechanisms were not measured in the present survey and should be treated as hypotheses for future research rather than explanations established by the data.

Poor self-rated health was associated with an increased use of teleconsultations, which is compatible with the role of these consultations in ongoing care, prescription renewal, and follow-up when an examination is not required. The lower estimate for residents in the largest cities was statistically significant only in Model 5 and should not be interpreted as evidence of mediation by perceived benefit. Swedish evidence found that urbanicity alone did not explain digital primary care use after adjustments, while socioeconomic disadvantage and morbidity remained relevant (22), indicating that geographic patterns may be context-specific.

Although the study did not measure community engagement, the results suggest that the implementation of telephone consultations should be monitored across socioeconomic groups and regions. Clear information for patients, support for those with limited digital access, and the continued availability of in-person visits may help reduce disparities in the use of teleconsultations. The uptake and outcomes should be monitored based on socioeconomic status and region, and these findings should be shared with the participating communities and decision-makers. These recommendations are consistent with the World Health Organization’s emphasis on equitable, person-centered digital health and with evidence that meaningful community participation depends on sustained organizational and community processes (34, 35). These are implementation priorities to be evaluated, rather than effects demonstrated by this cross-sectional study.

## 5 Limitations

Several limitations should be considered. First, the cross-sectional design did not establish temporality or causality; respondents who used telephone consultations may have formed more favorable estimates of the time saved, introducing the possibility of reverse causation and common method bias. Second, time savings were measured using a study-specific single item and broad categories that combined experienced and anticipated savings. Third, the outcome captured telephone consultations and should not be generalized to video or asynchronous care. Fourth, the survey did not measure digital literacy, device access, connectivity, trust, clinical indication, continuity with the primary care practice, or provider propensity to offer telephone consultations. Fifth, complete-case sample sizes differed across models, and the available outputs did not isolate the contribution of residence size controls, voivodeship indicators, and sample composition to the Model 5 coefficient change. Sixth, the analysis set included 54 respondents who reported needing primary care without contact; a sensitivity analysis restricted to respondents with actual contact would clarify the influence of this inclusion rule. Finally, the study did not measure community engagement, partnership processes, or knowledge translation mechanisms, so it cannot directly evaluate the mechanisms at the national level.

## 6 Conclusion

Reported or anticipated time savings were associated with the use of telephone consultations in the fully adjusted models, whereas no statistically significant associations were observed in the first four models. Lower household financial status was associated with decreased use of these services despite public funding. The findings support further equity-focused and community-informed evaluation of post-pandemic remote primary care; however, the cross-sectional design and specification-dependent results preclude causal conclusions. Taken together, the study reinforces the need to evaluate the sustained role of telephone consultations in relation to both the tangible benefits perceived by patients and the socioeconomic and regional conditions in which remote care is delivered. Telephone consultations may complement in-person primary care when clinically appropriate, but equitable implementation requires clear information, accessible support, and continued availability of face-to-face options for patients who need or prefer them. Future research should use longitudinal or linked data, conduct same-sample specification checks, and involve patients, community organizations, and primary care professionals in identifying barriers and adapting remote care pathways to local needs.

## Data Availability

The datasets presented in this article are not readily available because the contract agreement stipulated restrictions to share datasets. Requests to access the datasets should be directed to kbysze@sgh.waw.pl.
