# Supplementary material for: Reported time savings and telephone consultation use in polish primary care: a national random sample survey
Source: Front Public Health. 2026 Sep 25;14:1940990. doi: 10.3389/fpubh.2026.1940990 (PMC13649695; doi:10.3389/fpubh.2026.1940990)
Supplement: Supplementary file 1 [file Table_1.DOCX]

Supplementary Material

# 1 Supplementary Table S1 | Full logistic regression models of telephone-consultation use and reported time savings (odds ratios).

|  | **(1)** | **(2)** | **(3)** | **(4)** | **(5)** | **(6)** |
| --- | --- | --- | --- | --- | --- | --- |
| *Dependent variable: Used telephone consultation in primary care during past 12 months (Yes/No)* | | | | | | |
| **Reported time savings (ref: no savings)** |  |  |  |  |  |  |
| < 1 hour | 0.840 | 0.818 | 0.761 | 0.780 | 3.032*** | 3.154*** |
|  | (0.217) | (0.213) | (0.205) | (0.216) | (1.244) | (1.333) |
| 1–2 hours | 1.501 | 1.480 | 1.427 | 1.376 | 3.907*** | 4.134*** |
|  | (0.388) | (0.386) | (0.381) | (0.380) | (1.596) | (1.752) |
| 3 hours or more | 1.180 | 1.152 | 1.017 | 1.063 | 4.408*** | 4.866*** |
|  | (0.391) | (0.386) | (0.350) | (0.383) | (2.196) | (2.525) |
| **Self-rated health (ref: very good)** |  |  |  |  |  |  |
| Good |  | 0.880 | 0.980 | 1.073 | 1.290 | 1.154 |
|  |  | (0.165) | (0.209) | (0.235) | (0.338) | (0.308) |
| Fair |  | 0.896 | 1.241 | 1.650* | 1.238 | 1.146 |
|  |  | (0.189) | (0.336) | (0.479) | (0.421) | (0.397) |
| Poor |  | 1.204 | 2.189** | 3.495*** | 3.214** | 2.764** |
|  |  | (0.394) | (0.851) | (1.490) | (1.599) | (1.406) |
| Very poor |  | 0.166 | 0.290 | 0.345 | 0.368 | 0.415 |
|  |  | (0.184) | (0.338) | (0.427) | (0.476) | (0.532) |
| **Age group (ref: < 25 years)** |  |  |  |  |  |  |
| 25–34 |  |  | 1.124 | 0.642 | 0.498 | 0.549 |
|  |  |  | (0.513) | (0.567) | (0.505) | (0.566) |
| 35–44 |  |  | 0.909 | 0.526 | 0.427 | 0.525 |
|  |  |  | (0.417) | (0.464) | (0.429) | (0.537) |
| 45–54 |  |  | 0.790 | 0.511 | 0.452 | 0.578 |
|  |  |  | (0.368) | (0.454) | (0.458) | (0.596) |
| 55–64 |  |  | 0.798 | 0.668 | 0.712 | 0.959 |
|  |  |  | (0.373) | (0.600) | (0.729) | (1.001) |
| 65 and over |  |  | 0.495 | 0.526 | 0.590 | 0.723 |
|  |  |  | (0.240) | (0.499) | (0.644) | (0.806) |
| **Sex (ref: female)** |  |  |  |  |  |  |
| Male |  |  | 0.947 | 0.944 | 0.942 | 0.938 |
|  |  |  | (0.142) | (0.150) | (0.173) | (0.176) |
| **Marital status (ref: single)** |  |  |  |  |  |  |
| Informal partnership |  |  | 1.167 | 1.203 | 1.321 | 1.449 |
|  |  |  | (0.419) | (0.452) | (0.550) | (0.615) |
| Married |  |  | 1.936** | 1.771** | 1.406 | 1.414 |
|  |  |  | (0.511) | (0.493) | (0.431) | (0.441) |
| Other |  |  | 1.208 | 1.211 | 1.244 | 1.291 |
|  |  |  | (0.629) | (0.667) | (0.755) | (0.796) |
| **Children < 18 years (ref: no)** |  |  |  |  |  |  |
| Yes |  |  | 1.105 | 1.124 | 1.172 | 1.234 |
|  |  |  | (0.241) | (0.260) | (0.309) | (0.330) |
| **Education (ref: primary)** |  |  |  |  |  |  |
| Vocational |  |  |  | 0.451 | 0.769 | 0.663 |
|  |  |  |  | (0.235) | (0.476) | (0.423) |
| Secondary |  |  |  | 0.649 | 1.129 | 0.963 |
|  |  |  |  | (0.338) | (0.699) | (0.616) |
| Higher – medical |  |  |  | 0.650 | 0.779 | 0.569 |
|  |  |  |  | (0.401) | (0.569) | (0.429) |
| Higher – non-medical |  |  |  | 0.859 | 1.442 | 1.297 |
|  |  |  |  | (0.484) | (0.976) | (0.906) |
| **Financial situation (ref: very good)** |  |  |  |  |  |  |
| Rather good |  |  |  | 0.722 | 0.637 | 0.670 |
|  |  |  |  | (0.167) | (0.180) | (0.193) |
| Average |  |  |  | 0.416*** | 0.381*** | 0.424** |
|  |  |  |  | (0.116) | (0.133) | (0.151) |
| Rather poor |  |  |  | 0.202*** | 0.222*** | 0.243** |
|  |  |  |  | (0.100) | (0.128) | (0.146) |
| Very poor |  |  |  | 0.256 | 0.236 | 0.235 |
|  |  |  |  | (0.305) | (0.314) | (0.312) |
| **Employment status (ref: employed – contract)** |  |  |  |  |  |  |
| Civil/temporary contract |  |  |  | 0.415 | 0.756 | 0.705 |
|  |  |  |  | (0.232) | (0.504) | (0.478) |
| Self-employed |  |  |  | 0.549 | 0.701 | 0.699 |
|  |  |  |  | (0.224) | (0.331) | (0.332) |
| Unemployed |  |  |  | 2.891 | 5.309 | 4.167 |
|  |  |  |  | (2.863) | (6.462) | (5.237) |
| Retired (old-age pension) |  |  |  | 0.786 | 0.897 | 0.935 |
|  |  |  |  | (0.247) | (0.350) | (0.377) |
| Retired (disability pension) |  |  |  | 2.524 | 5.419** | 6.310** |
|  |  |  |  | (1.738) | (4.337) | (5.211) |
| Student |  |  |  | 0.560 | 0.311 | 0.376 |
|  |  |  |  | (0.527) | (0.346) | (0.426) |
| Homemaker |  |  |  | 1.523 | 1.801 | 1.850 |
|  |  |  |  | (0.956) | (1.292) | (1.339) |
| **Place of residence (ref: rural)** |  |  |  |  |  |  |
| Town < 20,000 inhabitants |  |  |  |  | 0.590 | 0.593 |
|  |  |  |  |  | (0.233) | (0.242) |
| City 20,000–100,000 |  |  |  |  | 1.227 | 1.337 |
|  |  |  |  |  | (0.295) | (0.330) |
| City 100,000–500,000 |  |  |  |  | 1.268 | 1.317 |
|  |  |  |  |  | (0.363) | (0.388) |
| City > 500,000 |  |  |  |  | 0.531** | 0.617 |
|  |  |  |  |  | (0.157) | (0.188) |
| **Perceived time savings – agree (ref: neutral)** |  |  |  |  |  |  |
| Strongly disagree |  |  |  |  |  | 1.643 |
|  |  |  |  |  |  | (0.931) |
| Rather disagree |  |  |  |  |  | 1.746 |
|  |  |  |  |  |  | (0.713) |
| Rather agree |  |  |  |  |  | 1.696* |
|  |  |  |  |  |  | (0.474) |
| Strongly agree |  |  |  |  |  | 1.913* |
|  |  |  |  |  |  | (0.649) |
| **Perceived money savings – agree (ref: neutral)** |  |  |  |  |  |  |
| Strongly disagree |  |  |  |  |  | 1.326 |
|  |  |  |  |  |  | (0.734) |
| Rather disagree |  |  |  |  |  | 0.575 |
|  |  |  |  |  |  | (0.204) |
| Rather agree |  |  |  |  |  | 0.782 |
|  |  |  |  |  |  | (0.200) |
| Strongly agree |  |  |  |  |  | 1.442 |
|  |  |  |  |  |  | (0.488) |

Odds ratios are reported, with robust standard errors clustered at the sampling-cluster level in parentheses. The analysis-eligible sample includes respondents who reported primary-care contact or a need for such contact during the previous 12 months. Models 1-4 use 789 complete cases; Models 5-6 use 736 complete cases. Reference category for reported time savings: no time savings. Model 5 adds residence-size controls and voivodeship indicators; Model 6 additionally adjusts for general perceptions that teleconsultations save time and money. * p < 0.10; ** p < 0.05; *** p < 0.01.

*Interpretation note: Model 5 simultaneously adds residence-size controls and voivodeship indicators and changes the complete-case sample. The coefficient change from Model 4 to Model 5 therefore cannot be attributed to voivodeship indicators alone without same-sample nested models and a joint test of the regional indicators.*
